# Metagenomics and Faecal Metabolomics Integrative Analysis towards the Impaired Glucose Regulation and Type 2 Diabetes in Uyghur-Related Omics

**DOI:** 10.1155/2019/2893041

**Published:** 2019-11-18

**Authors:** Rebiya Nuli, Jureti Azhati, Junxiu Cai, Aizhatiguli Kadeer, Bing Zhang, Patamu Mohemaiti

**Affiliations:** ^1^School of Public Health, Xinjiang Medical University, Urumqi 830011, China; ^2^College of Basic Medical Science, Xinjiang Medical University, Urumqi 830011, China; ^3^The People's Hospital of Xinjiang Uyghur Autonomous Region, Urumqi 830001, China; ^4^The Fifth Affiliated Hospital of Xinjiang Medical University, Urumqi 830000, China

## Abstract

**Objective:**

Gut microbiota and their metabolites play an important role in the development of type 2 diabetes mellitus (T2DM). This research was designed to study the relationship between gut microbiota and faecal metabolites of Uyghur newly onset T2DM and impaired glucose regulation (IGR) patients.

**Materials and Methods:**

A total of 60 different glycemic Uyghur subjects were enrolled and divided into T2DM, IGR, and normal glucose tolerance (NGT) groups. Metagenomics and LC-MS-based untargeted faecal metabolomics were employed. Correlations between bacterial composition and faecal metabolomics were evaluated.

**Results:**

We discovered that the composition and diversity of gut microbiota in newly onset T2DM and IGR were different from those in NGT. The *α*-diversity was higher in NGT than in T2DM and IGR; *β*-diversity analysis revealed apparent differences in the bacterial community structures between patients with T2DM, IGR, and NGT. LC-MS faecal metabolomics analysis discovered different metabolomics features in the three groups. Alchornoic acid, PE (14 : 0/20 : 3), PI, L-tyrosine, LysoPC (15 : 0), protorifamycin I, pimelic acid, epothilone A, 7-dehydro-desmosterol, L-lysine, LysoPC (14 : 1), and teasterone are the most significant differential enriched metabolites. Most of the differential enriched metabolites were involved in metabolic processes, including carbohydrate metabolism, starch and sucrose metabolism, phenylpropanoid biosynthesis, and biosynthesis of amino acids. Procrustes analysis and correlation analysis identified correlations between gut microbiota and faecal metabolites. Matricin was positively correlated with *Bacteroides* and negatively correlated with *Actinobacteria*; protorifamycin I was negatively correlated with *Actinobacteria*; epothilone A was negatively correlated with *Actinobacteria* and positively correlated with *Firmicutes*; PA was positively correlated with *Bacteroides* and negatively correlated with *Firmicutes*; and cristacarpin was positively correlated with *Actinobacteria*; however, this correlation relationship does not imply causality.

**Conclusions:**

This study used joint metagenomics and metabolomics analyses to elucidate the relationship between gut microbiota and faecal metabolites in different glycemic groups, and the result suggested that metabolic disorders and gut microbiota dysbiosis occurred in Uyghur T2DM and IGR. The results provide a theoretical basis for studying the pathological mechanism for further research.

## 1. Introduction

Diabetes mellitus (DM) is a globally prevalent chronic metabolic disease. The International Diabetes Federation (IDF) estimated that there were 451 million people with diabetes worldwide in 2017 [1]. Type 2 diabetes mellitus (T2DM) accounts for about 90% of diabetic cases. Impaired glucose regulation (IGR) is a prediabetic state, including impaired fasting glucose (IFG) and impaired glucose tolerance (IGT). Moreover, there was an estimated 374 million people with IGT in 2017 [1]. T2DM is the most important health problem in China [2, 3]. In the last few years, the prevalence rate of IGR patients in China has increased to 35.7% [4]. Xinjiang is the largest province located in the northwest of China with a diverse ethnic population. The crude prevalence of diabetes in the Chinese Uyghur population is 12.2% as well as the prevalence of prediabetes is 39.4% [4].

Researches on T2DM reported that the reduced gut microbiota diversity is one of the important environmental risk factors for metabolic disease [5] and is associated with biological metabolic markers [6]. However, the gut microbiome of Chinese Uyghur has not been well explored. Some studies have shown that compositional variations of the human microbiome from populations who have different geographic regions and dietary patterns were significant compared to the microbiomes from other previously studied populations [7].

A lot of numbers of bacterial metabolites are likely to have a significant impact on human physiology and disease development. Short-chain fatty acids (SCFAs) are currently the most studied bacterial products that have a beneficial effect on host health [8]. By integrating metagenomics and metabolomics information, we would better understand the interplay between gut microbiota and host metabolism. Therefore, metabolomics is the better tool to understand the complex metabolic interactions between gut microbes and their host [9].

To explore the potential characteristic metabolites that are associated with newly onset T2DM and IGR in sufferers, a nontargeted metabolomics technique is performed to discover potential faecal metabolites, and correlation analyses are applied to find relationship between the specific metabolites and gut microbiota composition. Metabolomics and metagenomics offer an effective approach for identifying metabolites, gut microbiota, and associated pathways that are crucial for understanding the mechanisms underlying metabolite changes during different glycemic stages. This study undergone in newly onset T2DM and IGR patients reduced the interaction effect of nationality, dietary habit, and antidiabetic drug on the gut microbiota. This study was also aimed at exploring possible pathways and gut microbiota metabolites which may play roles in regulating the mechanism of T2DM and IGR.

## 2. Materials and Methods

### 2.1. Subjects

A total of 60 different glycemic subjects (without specific diet preference) aged from 30 to 60 years were recruited from the First and Fifth Affiliated Hospital of Xinjiang Medical University. Among them, twenty subjects were newly diagnosed with T2DM according to the American Diabetes Association (ADA) 2014 criteria. Twenty subjects were grouped in newly diagnosed IGR. The control group comprised 20 normal glucose tolerance (NGT) subjects who were matched for age and gender to cases. The following exclusion criteria were applied in patients and control subjects: receipt of antidiabetic medicine (metformin, etc.), having antibiotic or drugs used to regulate intestinal flora (i.e., prebiotic, symbiotic, or probiotics), cardiovascular disease, kidney disease, cancer, pregnant women, lactating women, neurological impairments, and/or severe mental illness. Subjects who had pets at home were excluded. This study was approved by the First Affiliated Hospital of Xinjiang Medical University ethics committee. Informed consent was obtained from all participants.

### 2.2. Metagenomics Analysis

Midstream faecal samples were collected from all subjects in the morning and transported to the lab in dry ice and frozen at -80°C. Microbial DNA was extracted from a total of 60 frozen faecal samples using the QIAamp DNA Stool Mini Kit (Qiagen, Germany). The V3-V4 hypervariable region of the 16S rRNA was amplified with primers 338F (5′-ACTCCTACGGGAGGCAGCAG-3′) and 806R (5′-GGACTACHVGGGTWTCTAAT-3′) by the thermocycler PCR system (GeneAmp 9700, ABI, USA). The PCR reactions were conducted using the following program: 3 min of denaturation at 95°C, 27 cycles of 30 s at 95°C, 30 s for annealing at 55°C, 45 s for elongation at 72°C, and a final extension at 72°C for 10 min. PCR reactions were performed in triplicate 20 *μ*L mixture containing 4 *μ*L of 5×FastPfu buffer, 2 *μ*L of 2.5 mM dNTPs, 0.8 *μ*L of each primer (5 *μ*M), 0.4 *μ*L of FastPfu polymerase, and 10 ng of template DNA. The resulting PCR products were extracted from a 2% agarose gel and further purified using the AxyPrep DNA Gel Extraction Kit (Axygen Biosciences, Union City, CA, USA) and quantified using QuantiFluor™-ST (Promega, USA) according to the manufacturer's protocol. Purified amplicons were pooled in equimolar amounts and paired-end sequenced (2 × 300) on an Illumina MiSeq platform (Illumina, San Diego, USA) according to standard protocols by Major Bio-Pharm Technology Co. Ltd. (Shanghai, China) [10]. The abundance of bacteria at the phylum and genus levels was analyzed by using the JSD distance algorithm, and the optimal cluster *K* value was 2 (the highest CH index).

### 2.3. Untargeted Faecal Metabolomics Analysis

Frozen stool samples were thawed at 4°C. The weight of each sample from each group was approximately 60 mg. Metabolites were extracted by adding 600 *μ*L of methanol : water (2 : 1, *v*/*v*) and adding 20 *μ*L of internal standard (L-2-chlorophenylalanine, 0.3 mg/mL, methanol configuration), followed by homogenate. Ultrasonic crushing was performed at a low temperature for 10 min, followed by -20°C for 30 min. The samples were then centrifuged at 13,000 rpm, 4°C for 15 min, and 200 *μ*L supernatant was dried in a LC-MS vacuum centrifuge and analyzed by using liquid chromatograph-mass spectrometer (LC-MS) platform.

The platform for LC-MS analysis was Waters' UPLC-Q-TOF/MS. LC-MS was performed on an Ultimate 3000-Velos Pro system equipped with a binary solvent delivery manager and a sample manager, coupled with a LTQ Orbitrap Mass Spectrometer equipped with an electrospray interface (Thermo Fisher Scientific, USA). LC conditions were set as follows: ACQUITY BEH C18 column (100 mm × 2.1 mm i.d., 1.7 *μ*m; Waters, Milford, USA). The column was maintained at 45°C and separation was achieved using the following gradient: 5%B-20%B over 0-2 min, 20%B-60%B over 2-8 min, 60%B-100%B over 8-12 min, 100%B 2 min, and 14-14.5 min holding at 5%B at a flow rate of 0.40 mL/min, where B is acetonitrile (0.1% (*v*/*v*) formic acid) and A is aqueous formic acid (0.1% (*v*/*v*) formic acid) in the positive mode and B is acetonitrile (containing 5 mM ammonium formate) and A is water (containing 5 mM ammonium formate) in the negative mode. Injection volume was 3.00 *μ*L and column temperature was set at 45.0°C.

The mass spectrometric data were collected using a LTQ Orbitrap Mass Spectrometer equipped with an electrospray ionization (ESI) source operating in either the positive or negative ion mode. The capillary and source temperature was set at 500°C, with a desolvation gas flow of 90 L/h. Centroid data were collected from 50 to 1,000 m/z with a 30,000 resolution. The scan time and interval are 0.1 s and 0.02 s, respectively.

The quality control (QC) sample was prepared by mixing the extracts of all the samples in equal volume. Each QC has the same volume as the sample and was processed and detected in the same way as the analysis sample. In the process of instrument analysis, the QC sample was inserted into every eight samples to examine the stability and reproducibility of the entire analysis process. Raw data were normalized by the metabolomics processing software Progenesis QI (Waters Corporation, Milford, USA) to obtain the data matrix of retention time (RT), m/z data, and peak intensity.

### 2.4. Data Processing and Statistical Data Analysis

Baseline information analysis and metagenomics analyses were performed as described in our previous report [10]. Metabolomics analysis of normalized data by Excel 2007 (Microsoft, USA) processing, importing the normalized data matrix into the SIMCA-P+14.0 software package (Umetrics, Umea, Sweden), using unsupervised principal component analysis (PCA), and orthogonal partial least squares discriminant analyses (OPLS-DA) were performed to distinguish the overall differences in metabolic profiles between groups and to find differential metabolites between groups. OPLS-DA was performed, and 7-fold crossvalidation and response permutation testing were used to evaluate the robustness of the model. Multidimensional analyses of OPLS-DA and Student *t*-test were used to screen differential metabolites (variable important in projection (VIP) > 1, *P* value < 0.05, fold change < 0.8 or >1.2). The raw data was searched and identified by using the metabolomics processing software Progenesis QI (Waters Corporation, Milford, USA). The HMDB and KEGG databases were used to analyze the differential metabolites. In addition, all differentially abundant metabolites were queried against the online Kyoto Encyclopedia of Genes and Genomes (KEGG, http://www.kegg.jp/) and mapped to KEGG pathways. Enrichment analysis was performed to further explore the impact of differentially expressed metabolites and to analyze the internal relationships between differentially expressed metabolites. Only functional categories and pathways with *P* < 0.05 were considered to have significant enrichment. Procrustes analysis and Spearman correlation analysis were used to analyze the relationship between gut microbiota and changed faecal metabolites with the R vegan package. The correlation matrix between metabolites and the gut microbiota phylum and genus was generated by using the Pearson correlation coefficient.

## 3. Results

### 3.1. Patient Characteristics

No statistic differences were observed in age, sex, BMI, and blood lipid levels among the groups. Baseline characteristics of the study participants were shown in our previous study [10].

### 3.2. Metagenomics Findings

**Figure 1:**
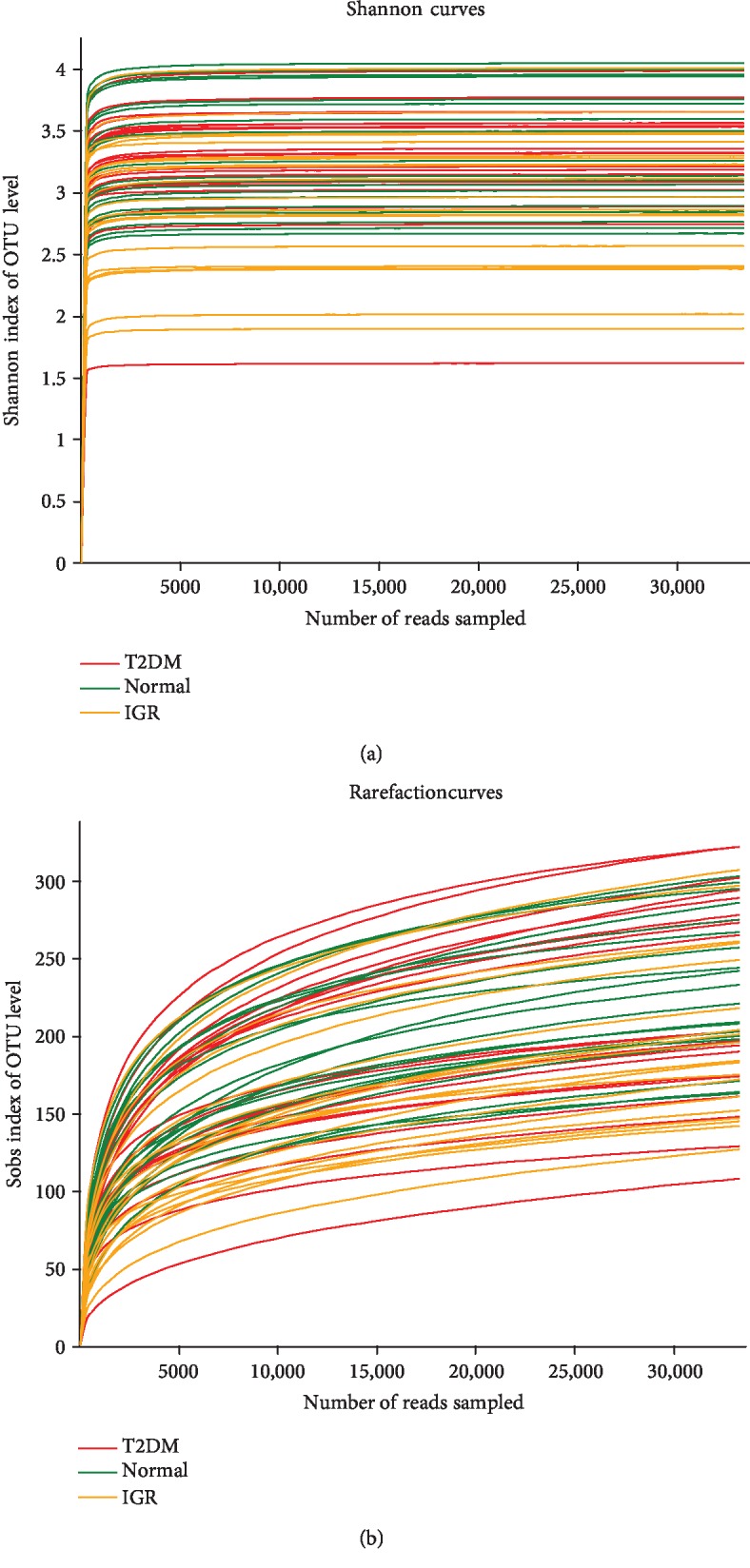
(a) Shannon-Wiener curves of the OTUs derived from the three groups. (b) Rarefaction curves of the OTUs derived from the three groups. OTU: operational taxonomic unit.

Based on the results of the OTU analysis, the Shannon-Wiener curve and rarefaction curve indicate that the sequencing depth was sufficient to explore the gut microbiota in three groups (Figures 1(a) and 1(b)).

**Figure 2:**
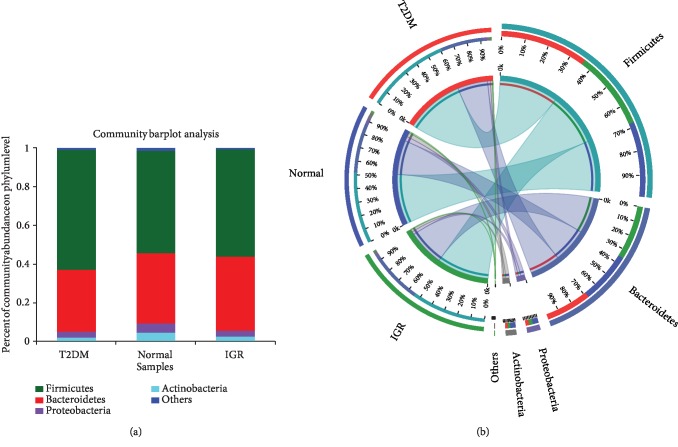
Bacterial richness distribution at the phylum level. (a) Community bar chart: different colours represent different bacterial phyla, and height of the colour represents the richness of bacterial phylum. (b) Circos sample-species relationship diagram: distribution of microbial community for each sample at the phylum level. The length of the ribbon from each phylum represents the relative abundance of that phylum in the sample.

At the phylum level, *Firmicutes*, *Bacteroidetes*, *Proteobacteria*, and *Actinobacteria* were dominant bacterial phyla in the three groups (Figure 2(a)). Among them, *Firmicutes* and *Bacteroidetes* had the highest abundance (Figure 2(b)). The *α*-diversity was highest in NGT, followed by T2DM and IGR.

**Figure 3:**
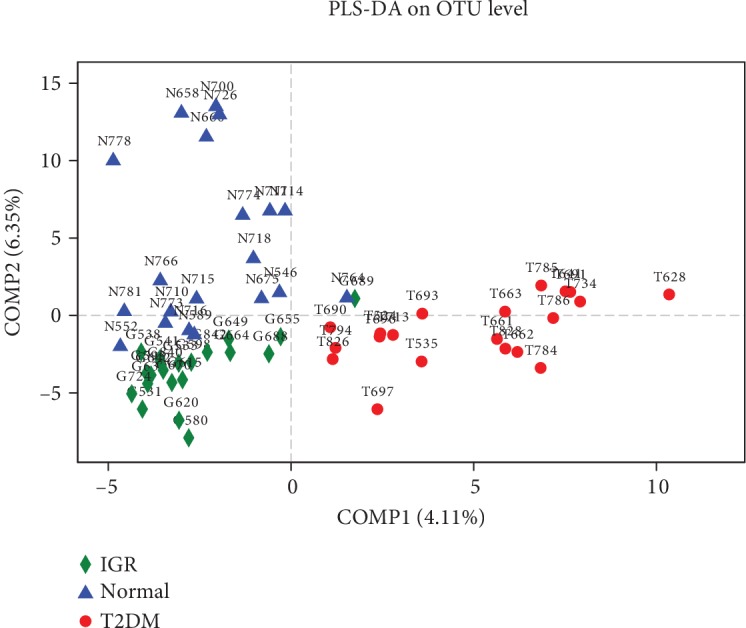
PLS-DA score plot based on OTU of microbial community in three groups. The blue triangles represent subjects with NGT and the red dots represent T2DM subjects. The green rhombus represents IGR subjects. Smaller distances between two points indicate greater similarity in microbial community structure between the two samples. PLS-DA: partial least squares discriminant analysis.

To view the similarities in gut bacterial community structures among patients with T2DM, IGR, and NGT, PLS-DA of *β*-diversity were performed according to the unweighted UniFrac distances. The results revealed apparent differences in the bacterial community structures among patients with T2DM, IGR, and NGT (Figure 3).

By mapping sequences to the Greengenes database, the functional gene contents of the gut microbiota were predicted by PICRUSt and were mapped on COG. The gut microbiota of the three groups showed 13 enriched COG functional orthologues, which were related to carbohydrate transport and metabolism; general function prediction only; amino acid transport and metabolism; replication, recombination, and repair; transcription; cell wall/membrane/envelope biogenesis; translation, ribosomal structure, and biogenesis; inorganic ion transport and metabolism; energy production and conversion; signal transduction mechanisms; coenzyme transport and metabolism; nucleotide transport and metabolism; defense mechanisms; and lipid transport and metabolism (Supplementary [Supplementary-material supplementary-material-1]). The analysis of microbial community structure and functions from the three groups revealed significant insights. Thus, to gain deeper insights into the metabolic activity of microbiomes from different glycemic groups, faecal metabolites were analyzed using a LC-MS-based metabolomics approach.

### 3.3. Metabolic Findings in Stool Samples

**Figure 4:**
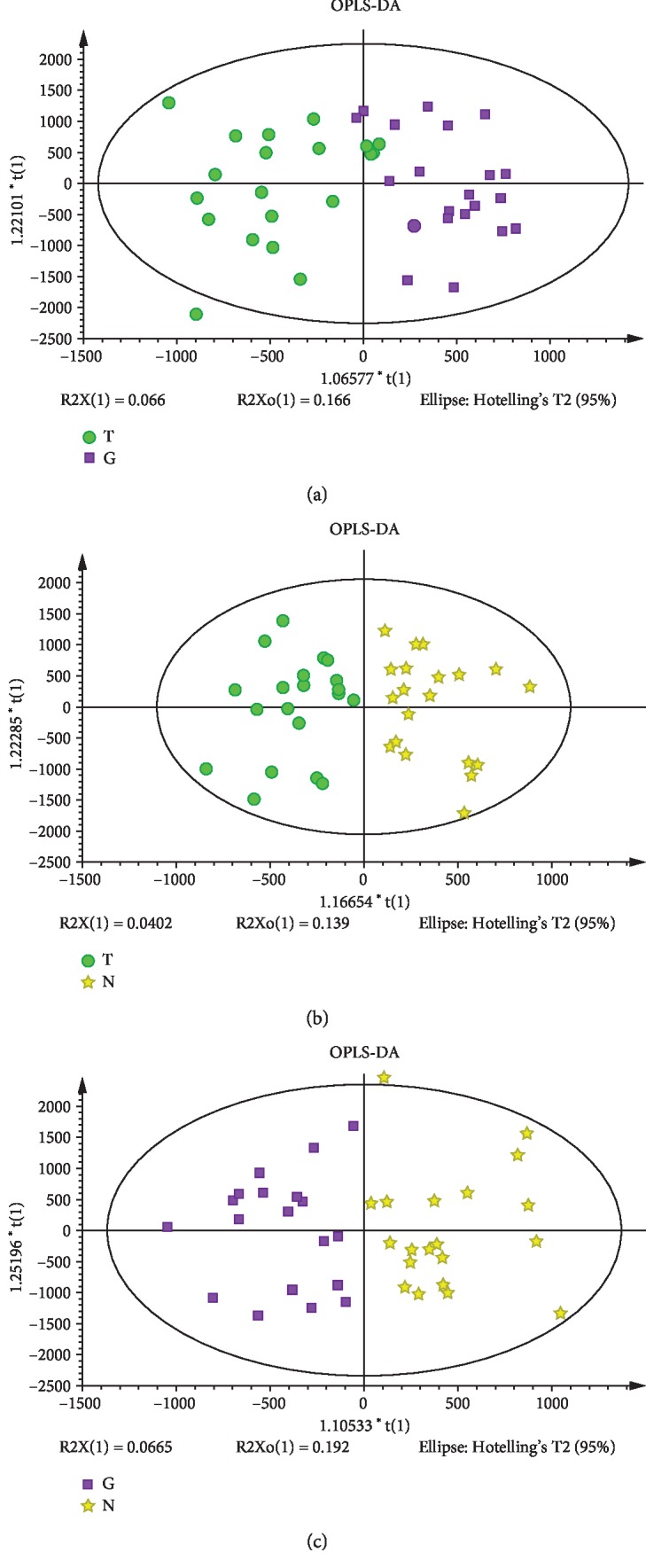
(a) Score plot of OPLS-DA in the T2DM and IGR groups; (b) score plot of OPLS-DA in the T2DM and NGT groups; (c) score plot of OPLS-DA in the IGR and NGT groups. Green circles represent the T2DM group, purple squares represent the IGR group, and yellow stars represent the NGT group.

LC-MS faecal metabolomics analysis discovered the different metabolomics features in the three groups. Both PCA and OPLS-DA score plots showed that there were significant differences between the T2DM and IGR groups, the T2DM and NGT groups, and the IGR and NGT groups, indicating that different glycemic statuses have different faecal metabolomics profiles (Figures 4(a)–4(c)). Permutation testing shows no overfitting data and validates the model of PLS-DA.

**Figure 5:**
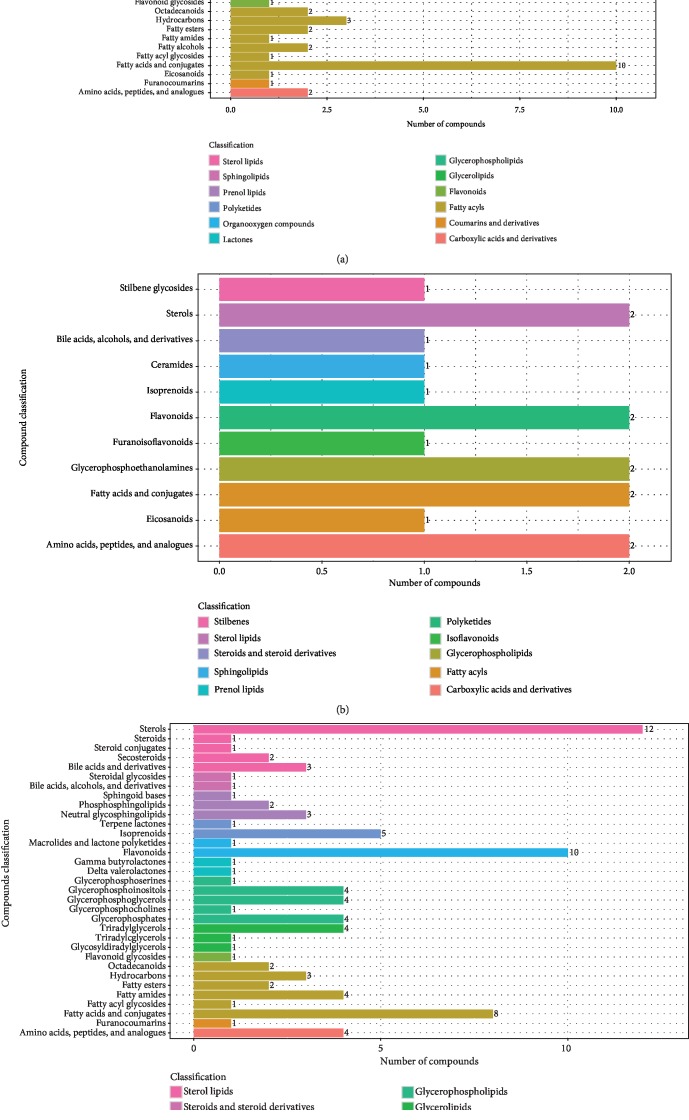
Classification of significantly varying metabolites (a) between the T2DM and IGR groups, (b) between the T2DM and NGT groups, and (c) between the IGR and NGT groups.

The significantly differential metabolites were selected based on the criteria of an OPLS-DA model VIP > 1 and a *P* value < 0.05. LC-MS metabolomics analysis discovered seventy-seven differentially enriched metabolites between T2DM and IGR; thirty-four of the metabolites were elevated in T2DM (fold change > 1.2) while forty-three of them were decreased (fold change < 0.8). The following metabolites were characterized: sterol lipid (*n* = 8), sphingolipids (*n* = 6), prenol lipids (*n* = 4), polyketides (*n* = 11), organooxygen compounds (*n* = 1), lactones (*n* = 1), glycerophospholipids (*n* = 10), glycerolipids (*n* = 5), flavonoids (*n* = 1), fatty acyls (*n* = 22), coumarins and derivatives (*n* = 1), and carboxylic acids and derivatives (*n* = 2) (Figure 5(a)). The levels of alchornoic acid, PI (13 : 0/22 : 1), 15-oxo-18Z-tetracosenoic acid, 2-oxo-docosanoic acid, and anhydrorhodovibrin were decreased in the newly diagnosed T2DM group with fold changes of 9.74, 5.97, 4.07, 3.51, and 3.27, respectively. It can be seen that the five characteristic metabolites with the highest fold change belong to the fatty acyl group (fatty acyls), glycerophospholipids, and anhydrorhodovibrin.

Sixteen significant variations in metabolites were detected between T2DM and NGT; six of them were increased and ten were decreased. The sixteen varying metabolites involved were stilbenes (*n* = 1), sterol lipids (*n* = 2), steroids and steroid derivatives (*n* = 1), sphingolipids (*n* = 1), prenol lipids (*n* = 1), polyketides (*n* = 2), isoflavonoids (*n* = 1), glycerophospholipids (*n* = 2), fatty acyls (*n* = 3), and carboxylic acids and derivatives (*n* = 2) (Figure 5(b)). PE (P-16 : 0/14 : 0) and 12,13-dihydroxy-11-methoxy-9-octadecenoic acid were all downregulated in the newly diagnosed T2DM group with fold changes at 3.88 and 2.65, respectively. It can be seen that the two characteristic metabolites with the highest change ratio belong to fatty acyls and glycerophospholipids.

In total, ninety-seven differentially presented metabolites that distinguished IGR from NGT were identified. Fifty-three metabolites were significantly upregulated and forty-four were significantly downregulated. The following ninety-seven metabolites were characterized: sterol lipids (*n* = 19), steroids and steroid derivatives (*n* = 2), sphingolipids (*n* = 6), prenol lipids (*n* = 6), polyketides (*n* = 11), lactones (*n* = 2), glycerophospholipids (*n* = 14), glycerolipids (*n* = 6), flavonoids (*n* = 1), fatty acyls (*n* = 20), coumarins and derivatives (*n* = 1), and carboxylic acids and derivatives (*n* = 4) (Figure 5(c)). The levels of PI (12 : 0/19 : 0), PA (20 : 5/22 : 0), and PI (12 : 0/22 : 4) were upregulated in the IGR group with fold changes at 5.38, 4.68, and 3.19, respectively. It can be seen that the three characteristic metabolites with the highest change ratio belong to glycerophospholipids.

Differentially expressed metabolites were annotated by online databases HMDB, KEGG, and LIPID MAPS. And a total of eleven significant metabolites (L-tyrosine; LysoPC (15 : 0); protorifamycin I; pimelic acid; anhydrorhodovibrin; epothilone A; matricin; bacteriohopane-32,33,34,35-tetrol; 5 alpha,6 beta-dihydroxycholestanol; cytochalasin A; and (6S)-dehydrovomifoliol) from these 77 differentially enriched metabolites between T2DM and IGR could be annotated (Supplementary [Supplementary-material supplementary-material-1]). A total of five significant metabolites (PE (14 : 0/20 : 3), 7-dehydro-desmosterol, cristacarpin, piceid, and gamma-glutamylglutamine) from these 16 different metabolites between T2DM and NGT could be annotated while nineteen metabolites were annotated from the IGR and NGT groups (Supplementary Tables [Supplementary-material supplementary-material-1] and [Supplementary-material supplementary-material-1]).

**Figure 6:**
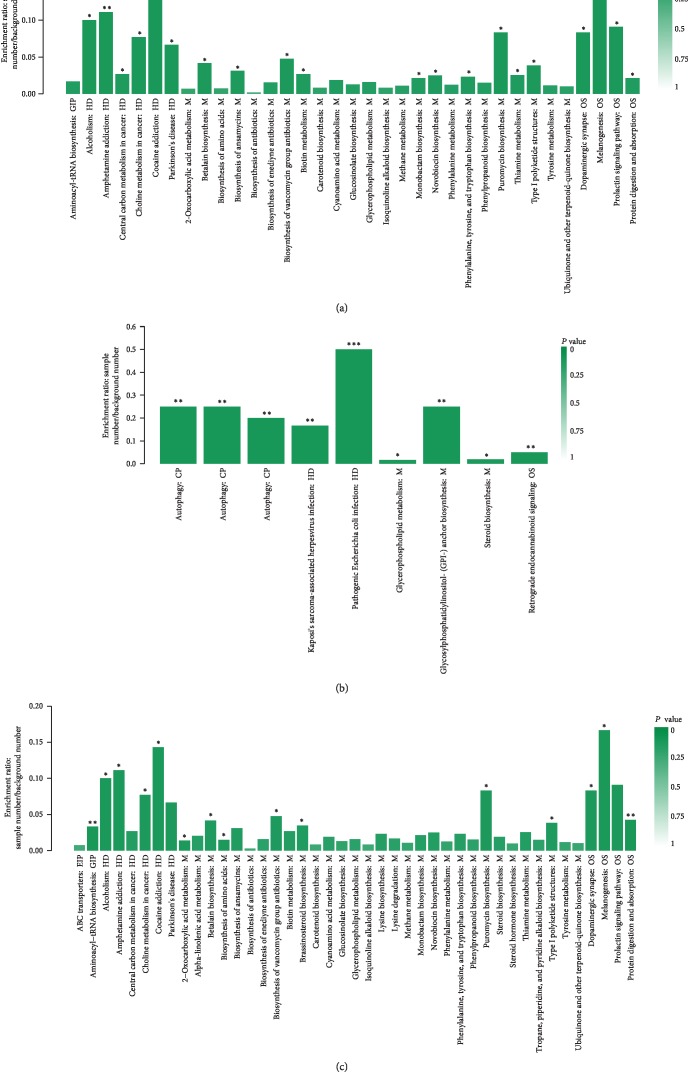
Metabolic pathway enrichment study of differentially presented metabolites between (a) the T2DM and IGR groups, (b) the T2DM and NGT groups, and (c) the IGR and NGT groups.

The KEGG database was used to analyze the differential metabolites. L-Tyrosine, LysoPC (15 : 0), protorifamycin I, pimelic acid, epothilone A, L-lysine, LysoPC (14 : 1), teasterone, PE (14 : 0/20 : 3), and 7-dehydro-desmosterol are involved in significant pathways. Most of them were involved in metabolism and human disease. We submitted the differential metabolites to the KEGG website for the analysis of relevant pathways. Analysis of metabolite pathways suggested that the following pathways were significantly changed in T2DM and IGR patients compared with NGT: phenylalanine, tyrosine, and tryptophan biosynthesis pathways, puromycin biosynthesis, vancomycin antibiotic biosynthesis, amino acid biosynthesis, glycosylphosphatidylinositol- (GPI-) anchored biosynthesis, steroidal biosynthesis, and glycerophospholipid metabolism (Figures 6(a)–6(c)).

To explore gut flora species significantly associated with the identified potential metabolites, integrated analysis of metabolomics and metagenomics was performed for the three groups.

### 3.4. Integrated Analysis of Gut Microbiota and Faecal Metabolomics

**Figure 7:**
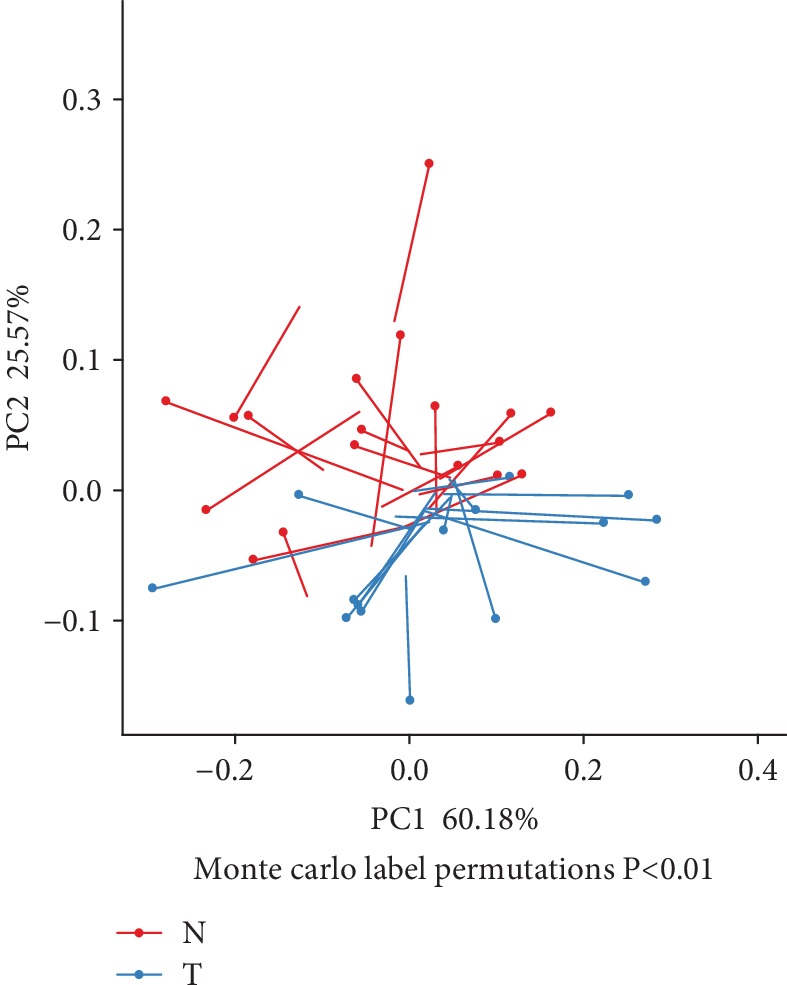
Procrustes analysis to assess the consistency of gut microbiome and faecal metabolomics profile data. Note: one dot and one line with an arrow represent one sample. The blue dots and blue lines represent subjects with T2DM, and the red dots and red lines represent NGT subjects. The dots represent oral microbiota compositions. Shorter lengths of the lines indicate greater consistency of the two datasets.

Firstly, a Procrustes analysis was performed to assess the consistency of the data from the gut microbiome and faecal metabolomics profiling; the results showed that the similarity between the two datasets was low although significance was found between the T2DM and NGT groups (*P* < 0.01, Figure 7).

Next, we analyzed possible correlations between altered faecal metabolites and microbial genera based on Spearman's correlation.

#### 3.4.1. Correlation Study of Gut Microbiota and Faecal Metabolomics between T2DM and IGR Groups

Data indicated that faecal decreased metabolites such as matricin, protorifamycin I, and epothilone A were negatively correlated with *Actinobacteria*, matricin and PA were positively correlated with *Bacteroides*, epothilone A was positively correlated with *Firmicutes*, and PA was negatively correlated with *Firmicutes* (Supplementary [Supplementary-material supplementary-material-1]).

On the genus level, *Coprococcus_3*, *Blautia*, *Subdoligranulum*, *Faecalibacterium*, and *Ruminococcus_torques_group* were closely associated with the accumulation of 6 faecal metabolites (Supplementary [Supplementary-material supplementary-material-1]).

#### 3.4.2. Correlation Study of Gut Microbiota and Faecal Metabolomics between T2DM and NGT Groups

The faecal decreased metabolite cristacarpin was positively correlated with *Actinobacteria* (*r* = 0.38, *P* < 0.05) (Supplementary [Supplementary-material supplementary-material-1]).

*Ruminococcaceae_UCG–005*, *Lachnospiraceae_NK4A136_group*, *Bifidobacterium*, *Parabacteroides*, *Bacteroides*, *Intestinibacter*, and *Subdoligranulum* were correlated with 4 faecal metabolites (Supplementary [Supplementary-material supplementary-material-1]).

#### 3.4.3. Correlation Study of Gut Microbiota and Faecal Metabolomics between IGR and NGT Groups

The faecal increased metabolite epothilone A was positively correlated with *Firmicutes* (*r* = 0.37, *P* < 0.05); the level of faecal decreased metabolite 3-O-(beta-D-glucopyranosyl-(1->6)-beta-D-glucopyranosyl) was positively correlated with *Bacteroides* and negatively correlated with *Firmicutes* (Supplementary [Supplementary-material supplementary-material-1]).

On the genus level, *Subdoligranulum*, *Eubacterium_coprostanoligenes_group*, *Lachnospiraceae_NK4A136_group*, *Eubacterium_rectale_group*, *Ruminococcus_torques_group*, *Butyricicoccus*, *Lachnospiraceae_ND3007_group*, *Fusicatenibacter*, *Coprococcus_1*, *Eubacterium_hallii_group*, *Lachnospiraceae_NC2004_group*, *Bacteroides*, *Eubacterium_rectale_group*, *Lachnoclostridium*, *Lachnospiraceae_NK4A136_group*, *Fusicatenibacter*, *Eubacterium_hallii_group*, *Faecalibacterium*, *Fusicatenibacter*, and *Bacteroides* were correlated with 14 faecal metabolites (Supplementary [Supplementary-material supplementary-material-1]).

In summary, by combining the association of gut microbiota phylum and genus with faecal metabolites, this study discovered that *Firmicutes* and their associated metabolites L-tyrosine, protorifamycin I, epothilone A, and L-lysine were involved in the development of IGR and T2DM in this population.

## 4. Discussions

This is the first integrative study report that applied high-throughput sequencing of microbial diversity and LC-MS-based metabolomics approach to study the gut microbiota diversity and faecal metabolic variations in Chinese Uyghur newly diagnosed T2DM and IGR patients. In our previous study, we reported that gut microbiota diversity of T2DM and IGR is different from that of a normal healthy group [10]. Moreover, these gut microbiota were associated with changes in several metabolomics profiles [10]. Different faecal metabolic profiles were discovered among the three groups, and some of them correlated with some bacteria, indicating that T2DM not only disturbed the gut microbiota at the abundance level but also substantially altered the faecal metabolomics profile related to the gut microbiome, resulting in disturbances in host metabolite homeostasis. Strict inclusion criteria were used; all of the Chinese Uyghur subjects in this study were Urumqi citizens. T2DM patients were newly diagnosed, without using any kind of antidiabetes medicine. Since antibiotics had influence on the gut microbiota diversity [11], subjects who used antibiotics in the previous month were excluded. Meanwhile, subjects under medication for hypertension, patients prescribed lipid-lowering drugs, and patients with cardiovascular disease history, special diet, dietary supplement use, and mental problems were all excluded. The main regulator of the gut microbiota includes age, ethnicity, diet, and immunity [12]. Compound factors were controlled in these 60 subjects. This study tried to achieve a balance within groups and tried to reduce the impact of other confounding factors on the gut microbiota and faecal metabolites.

It was reported that a stable and diverse gut microbiome is vital for human health; changes in the composition of gut microbial flora were associated with T2DM [13]. Gut microbiota composition and abundance were altered in different glycemic stages. The dominant phyla (*Firmicutes*, *Bacteroidetes*, *Proteobacteria*, and *Actinobacteria*) in this study were highly similar to those described previously [14, 15]. It was reported that two main bacterial phyla, *Firmicutes* and *Bacteroidetes*, are involved in the host metabolism and fat accumulation in T2DM patients [16]. Previous studies identified a higher amount of *Firmicutes* and lower amounts of *Bacteroidetes* and *Proteobacteria* phyla in T2DM patients [17].

Gut microbiota can directly participate in the metabolism of protein, fat, and carbohydrate and also take part in the metabolism of energy and biochemical metabolism of bile acid and bilirubin. The diversity and dynamic equilibrium of gut microbiota are very important for pathogen invasion and multiplication. Altered gut microbiota of IGR and T2DM affects the process of host metabolism and leads to the change of microbial metabolites. Both of gut microbiota and their metabolites have an effect on the host metabolism. In recent years, faecal metabolomics has increasingly gained attention and has shown remarkable results in characterizing microbial metabolic functions. The faecal samples are easy to access and provide a noninvasive sample matrix to study the metabolic activity of the host, the microbes, and their cometabolism [18]. Faecal can reflect the pathogenic change of the digestive system. So, studying the change of faecal metabolites in diabetic patients is useful to understand the pathogenic mechanism of the unbalanced gut microbiota in the development of IGR and T2DM.

There is a clear separation of faecal metabolomics profile in different glycemic stages. It was identified that several significantly abundant metabolites are associated with phenylalanine, tyrosine, tryptophan biosynthesis pathways, puromycin biosynthesis, vancomycin antibiotic biosynthesis, amino acid biosynthesis, glycosylphosphatidylinositol- (GPI-) anchored biosynthesis, steroidal biosynthesis, and glycerophospholipid metabolism.

In this study, significantly changed metabolites L-tyrosine, LysoPC, protorifamycin I, pimelic acid, epothilone A, PE, 7-dehydro-desmosterol, L-lysine, and teasterone were involved in the significant pathways. L-Tyrosine is one of the standard amino acids that are useful by cells to synthesize proteins. This study disclosed that in Chinese adults, tyrosine > 46 *μ*mol/L in plasma was associated with increased odds of T2DM, which was contingent upon low HDL-C [19]. Previous studies indicated that insulin resistance was connected with metabolism of tyrosine; insulin signalling pathways might be inhibited by elevated tyrosine levels, which is related to the development of T2DM; and it is suggested that the altered level of tyrosine might reflect the degree of inflammation in diabetes or prediabetes [20–23]. The tyrosine level is associated with the risk of T2DM in different ethnic peoples, and the relationship was robust by ethnicity and study designs [24, 25]. In this research, L-tyrosine in stool was upregulated in T2DM patients compared to IGR and was downregulated in IGR compared to NGT.

LysoPC (15 : 0) and LysoPC (14 : 1 (9Z)) are two kinds of lysophosphatidylcholines (LPC) which belong to glycerophospholipids, involved in choline metabolism in cancer and the glycerophospholipid metabolism pathway. LysoPC resulted from the partial hydrolysis of phosphatidylcholines, which removes one of the fatty acid groups; LysoPC can activate endothelial cells during early atherosclerosis and can stimulate phagocyte recruitment when they were released by apoptotic cells [26–28]. LPCs are the major components of ox-LDL which play dual functions in the cardiovascular disease.

This study indicated that protorifamycin I (which belongs to phenylpropanoids and polyketides) is enriched in the biosynthesis of ansamycins and biosynthesis of antibiotics.

Pimelic acid belongs to fatty acid, enriched in metabolic pathways in this study. Derivatives of pimelic acid are involved in the biosynthesis of the amino acid called lysine. It was reported that pimelic acid originating from the fatty acid synthesis pathway is a bona fide precursor of biotin in *Bacillus subtilis* [29].

PE (14 : 0/20 : 3) is one kind of glycerophosphoethanolamines. Phosphatidylethanolamine (PE) is class of phospholipids found in biological membranes; it can be found in all living organism. Together with phosphatidylcholine (PC), phosphatidylserine (PS), and phosphatidylinositol (PI), PE represents the backbone of most biological membranes. PE is the second-most abundant phospholipid in mammalian membranes ranging from 20 to 50% that positively regulate autophagy and longevity [30]. Phosphatidylethanolamines in food break down to form phosphatidylethanolamine-linked Amadori products as a part of the Maillard reaction [31]. These products accelerate membrane lipid peroxidation, causing oxidative stress to cells that come in contact with them [32]. Oxidative stress is known to cause several diseases. Significant levels of Amadori-phosphatidylethanolamine products have been found in a wide variety of food such as chocolate, soybean milk, infant formula, and other processed food. The level of Amadori-phosphatidylethanolamine products is higher in foods with high lipid and sugar concentrations that have high temperatures in processing [31]. Additional studies indicated that Amadori-phosphatidylethanolamine may play a role in vascular disease. It acts as the mechanism by which diabetes can increase the incidence of cancer and potentially play a role in other diseases as well [32, 33]. Amadori-phosphatidylethanolamine has shared a higher plasma concentration in diabetic patients than healthy people, indicating it may play a role in the development of the disease or be a product of the disease [34]. In this study, PE was upregulated in the stool of the newly diagnosed T2DM group.

7-Dehydro-desmosterol belongs to sterols, which are enriched in steroid biosynthesis pathways and metabolic pathways. Sterols are a subgroup of the steroids and an important class of organic molecules. They occur naturally in plants, animals, and fungi, and can also be produced by some bacteria. Bacterial sterol structure genomes were found from five phyla (*Bacteroides*, *Cyanobacteria*, *Planctomycetes*, *Proteobacteria*, and *Verrucomicrobia*) and also from uncultured bacteria [35].

L-Lysine is one of the nine essential amino acids in humans. L-Lysine was downregulated in IGR compare to that in NGT. Lysine frequently plays an important role in protein structure. Lysine has also been implicated to play a key role in other biological processes including structural proteins of connective tissues, calcium homeostasis, and fatty acid metabolism. Since lysine is essential for humans, and the human body cannot synthesize, it must be obtained from the diet. Most commonly, lysine deficiency is seen in non-Western societies and manifests as protein-energy malnutrition, which has profound and systemic effects on the health of the individual [36]. Due to its importance in several biological processes, a lack of lysine can lead to several disease states including defective connective tissues, impaired fatty acid metabolism, anemia, and systemic protein-energy deficiency. In contrast, an overabundance of lysine, caused by ineffective catabolism, can cause severe neurological issues [37]. Chen et al. reported lysine modifications as molecular markers in the diagnosis and treatment of cancer [38]. The study showed 12 differentiated microbes at the genus level in response to dietary lysine restriction in a pig model; at the phylum level, lysine restriction could enhance abundances of *Actinobacteria*, *Saccharibacteria*, and *Synergistetes*, suggesting that long-term lysine restriction from piglets to finishing pigs affected the amino acid metabolism, which might be associated with gut microbiota [39, 40].

This study concluded that differentially presented metabolites were mainly linked with the glycerophospholipid and sphingolipid metabolism pathway. Sphingolipid metabolism is important in the regulation of inflammatory signalling pathways, and dietary sphingolipids appear to influence inflammation-related chronic diseases by altering gut microbiota [41].

A previous study suggested that around 30% of metabolites detected in the human body originated from microbiota. In this study, it was showed a corelationship between the gut microbiota and faecal metabolites; however, the relationship between faecal metabolites and gut microbiota is purely correlative without further controlled experiments; large-sample, prospective studies should be performed to address any causal relationships.

In conclusion, this work had demonstrated altered gut microbiota and faecal metabolites in the Uyghur T2DM and IGR patients compared with healthy normal controls through a LC-MS-based metabolomics and metagenomics study. The sample size of this study was limited, so a larger number of samples are needed for population-based validation. The underlying mechanism regulating correlation between gut microbiota and metabolites like L-tyrosine, protorifamycin I, epothilone A, and L-lysine in IGR and T2DM incidents could be further investigated. However, there is no reliable way for early diagnosis and prevention of T2DM and IGR; so far, the development of novel microbial markers for early diagnosis of T2DM and IGR is urgently needed.

## Data Availability

The data used to support the findings of this study are available from the corresponding author upon request.

## Additional Points

*Highlights*. This study integrated the metagenomics and faecal metabolomics study in Chinese Uyghur newly diagnosed T2DM and IGR patients, in order to provide a theoretical basis for the study of the pathological mechanism of gut microbiota on T2DM.

## Supplementary Materials

Supplementary Materials Supplementary Table 1: annotated differentially enriched metabolites between the newly diagnosed T2DM and IGR groups. Supplementary Table 2: annotated differentially enriched metabolites between the newly diagnosed T2DM and NGT groups. Supplementary Table 3: annotated differentially enriched metabolites between the IGR and NGT groups. Supplementary Figure 1: box plot of COG function classification. Microbial sequences mapped by the Greengenes database; the functional gene contents of the gut microbiota were predicted by PICRUSt and were mapped on COG. Supplementary Figure 2: correlation heat map of differentially enriched metabolites and gut microbiota phylum between the newly diagnosed T2DM and IGR groups (a); correlation heat map of differentially enriched metabolites and gut microbiota phylum between the newly diagnosed IGR and NGT groups (b). Note: each column in the graph represents a metabolite, each row represents a microbiota phylum, the colour in the graph indicates the Pearson coefficient between the bacterial phylum and the metabolite, and the red colour indicates positive correlation. The green colour is representative of negative correlation. The darker colour indicates the greater the correlation. ∗ represents a significant correlation (*P* < 0.05). Supplementary Figure 3: correlation heat map of differentially enriched metabolites and gut microbiota genus between the newly diagnosed T2DM and IGR groups (a); correlation heat map of differentially enriched metabolites and gut microbiota genus between the newly diagnosed IGR and NGT groups (b). Note: each column in the graph represents a metabolite, each row represents a microbiota genus, the colour in the graph indicates the Pearson coefficient between the bacterial genus and the metabolite, and the red colour indicates positive correlation. The green colour is representative of negative correlation. The darker colour indicates the greater the correlation. ∗ represents a significant correlation (*P* < 0.05). Supplementary Figure 4: correlation heat map of differentially enriched metabolites and gut microbiota phylum level between the newly diagnosed T2DM and NGT groups (a); correlation heat map of differentially enriched metabolites and gut microbiota genus level between the newly diagnosed T2DM and NGT groups (b).
