# Supplementary material for: Metagenomics and Faecal Metabolomics Integrative Analysis towards the Impaired Glucose Regulation and Type 2 Diabetes in Uyghur-Related Omics
Source: J Diabetes Res. 2019 Nov 18;2019:2893041. doi: 10.1155/2019/2893041 (PMC6885810; doi:10.1155/2019/2893041)
Supplement: Supplementary Materials — Supplementary Table 1: annotated differentially enriched metabolites between the newly diagnosed T2DM and IGR groups. Supplementary Table 2: annotated differentially enriched metabolites between the newly diagnosed T2DM and NGT groups. Supplementary Table 3: annotated differentially enriched metabolites between the IGR and NGT groups. Supplementary Figure 1: box plot of COG function classification. Microbial sequences mapped by the Greengenes database; the functional gene contents of the gut microbiota were predicted by PICRUSt and were mapped on COG. Supplementary Figure 2: correlation heat map of differentially enriched metabolites and gut microbiota phylum between the newly diagnosed T2DM and IGR groups (a); correlation heat map of differentially enriched metabolites and gut microbiota phylum between the newly diagnosed IGR and NGT groups (b). Note: each column in the graph represents a metabolite, each row represents a microbiota phylum, the colour in the graph indicates the Pearson coefficient between the bacterial phylum and the metabolite, and the red colour indicates positive correlation. The green colour is representative of negative correlation. The darker colour indicates the greater the correlation. ∗ represents a significant correlation (P < 0.05). Supplementary Figure 3: correlation heat map of differentially enriched metabolites and gut microbiota genus between the newly diagnosed T2DM and IGR groups (a); correlation heat map of differentially enriched metabolites and gut microbiota genus between the newly diagnosed IGR and NGT groups (b). Note: each column in the graph represents a metabolite, each row represents a microbiota genus, the colour in the graph indicates the Pearson coefficient between the bacterial genus and the metabolite, and the red colour indicates positive correlation. The green colour is representative of negative correlation. The darker colour indicates the greater the correlation. ∗ represents a significant correlation (P < 0.05) [file 2893041.f1.pdf]

Supplementary Table 1: Annotated differentially enriched metabolites between the newly diagnosed T2DM and IGR groups

Supplementary Table 2: Annotated differentially enriched metabolites between the newly diagnosed T2DM and NGT groups

Supplementary Table 3: Annotated differentially enriched metabolites between the IGR and NGT groups

Supplementary Figure 1: Box plot of COG function classification. Microbial sequences mapped by Greengenes database, the functional gene contents of the gut microbiota were predicted by PICRUSt and were mapped on COG.

Supplementary Figure 2: Correlation heatmap of differentially enriched metabolites and gut microbiota phylum between the newly diagnosed T2DM and IGR group (a); Correlation heatmap of differentially enriched metabolites and gut microbiota phylum between the newly diagnosed IGR and NGT group (b)
Note: Each column in the graph represents a metabolite, each row represents a microbiota phylum, the colour in the graph indicates the Pearson coefficient between the bacterial phylum and the metabolite, and the red colour indicates positive correlation. The green colour is representative negative correlation. The darker colour indicate the greater the correlation. * is represents a significant correlation ($P$ <0.05).

Supplementary Figure 3: Correlation heatmap of differentially enriched metabolites and gut microbiota genus between the newly diagnosed T2DM and IGR group (a); Correlation heatmap of differentially enriched metabolites and gut microbiota genus between the newly diagnosed IGR and NGT group (b).
Note: Each column in the graph represents a metabolite, each row represents a microbiota genus, the colour in the graph indicates the Pearson coefficient between the bacterial genus and the metabolite, and the red colour indicates positive correlation. The green colour is representative negative correlation. The darker colour indicate the greater the correlation. * is represents a significant correlation ($P$ <0.05).

Supplementary Figure 4: Correlation heatmap of differentially enriched metabolites and gut microbiota phylum level between the newly diagnosed T2DM and NGT group (a), Correlation heatmap of differentially enriched metabolites and gut microbiota genus level between the newly diagnosed T2DM and NGT group (b).

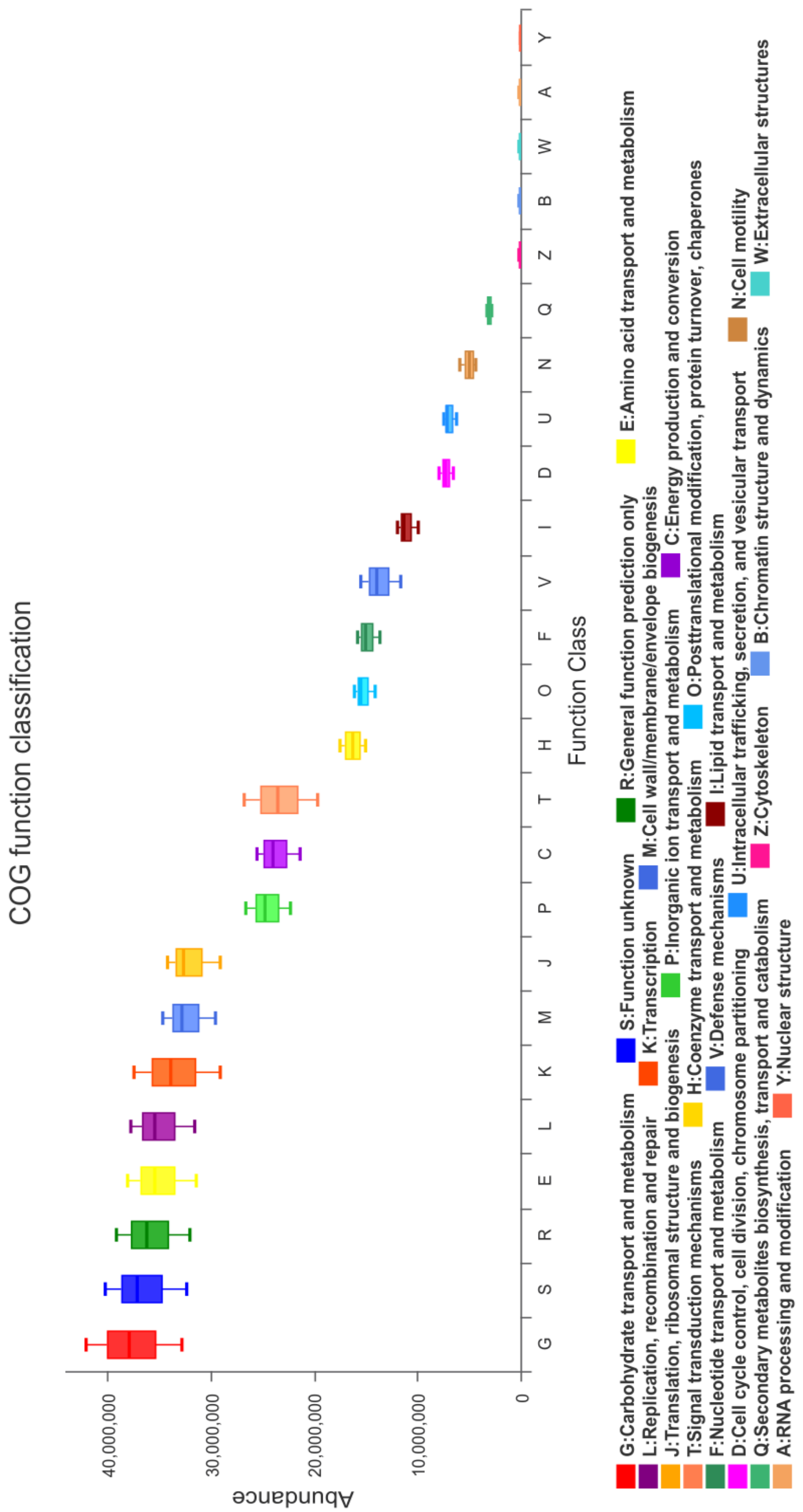


Supplementary Figure 1: Box plot of COG function classification. Microbial sequences mapped by Greengenes database, the functional gene contents of the gut microbiota were predicted by PICRUSt and were mapped on COG.

a

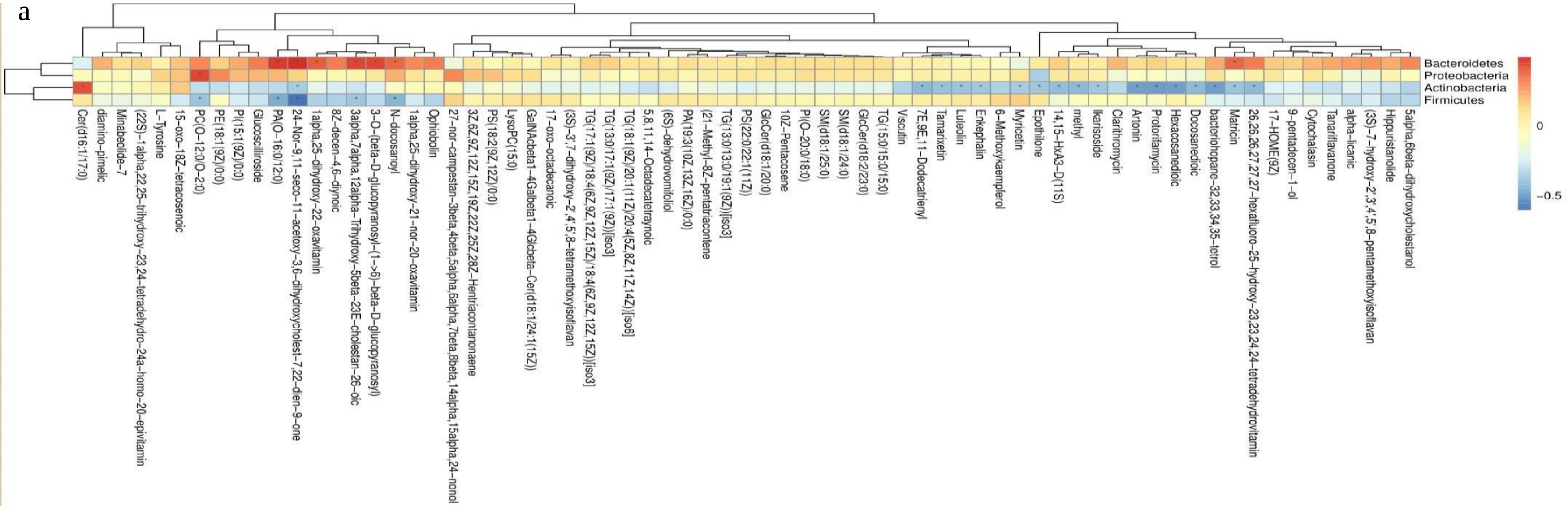
Bacteroidetes
Proteobacteria
Actinobacteria
Firmicutes
0
−0.5
Cer(d16:1/17:0)
diamino−pimelic
Minabeolide−7
(22S)−1alpha,22,25−trihydroxy−23,24−tetradehydro−24a−homo−20−epivitamin
L−Tyrosine
15−oxo−18Z−tetracosenoic
PC(O−12:0/O−2:0)
PE(18:1(9Z)/0:0)
PI(15:1(9Z)/0:0)
Glucoscilliroside
PA(O−16:0/12:0)
24−Nor−9,11−seco−11−acetoxy−3,6−dihydroxycholest−7,22−dien−9−one
1alpha,25−dihydroxy−22−oxavitamin
8Z−decen−4,6−diynoic
3alpha,7alpha,12alpha−Trihydroxy−5beta−23E−cholestan−26−oic
3−O−(beta−D−glucopyranosyl−(1−>6)−beta−D−glucopyranosyl)
N−docosanoyl
1alpha,25−dihydroxy−21−nor−20−oxavitamin
Ophiobolin
27−nor−campestan−3beta,4beta,5alpha,6alpha,7beta,8beta,14alpha,15alpha,24−nonol
3Z,6Z,9Z,12Z,15Z,19Z,22Z,25Z,28Z−Hentriacontanonaene
PS(18:2(9Z,12Z)/0:0)
LysoPC(15:0)
GalNAcbeta1−4Galbeta1−4Glcbeta−Cer(d18:1/24:1(15Z))
17−oxo−octadecanoic
(3S)−3',7−dihydroxy−2',4',5',8−tetramethoxyisoflavan
TG(17:1(9Z)/18:4(6Z,9Z,12Z,15Z)/18:4(6Z,9Z,12Z,15Z))[iso3]
TG(13:0/17:1(9Z)/17:1(9Z))[iso3]
TG(18:1(9Z)/20:1(11Z)/20:4(5Z,8Z,11Z,14Z))[iso6]
5,8,11,14−Octadecatetraynoic
(6S)−dehydrovomifoliol
PA(19:3(10Z,13Z,16Z)/0:0)
(21−Methyl−8Z−pentatriacontene
TG(13:0/13:0/19:1(9Z))[iso3]
PS(22:0/22:1(11Z))
GlcCer(d18:1/20:0)
10Z−Pentacosene
PI(O−20:0/18:0)
SM(d18:1/25:0)
SM(d18:1/24:0)
GlcCer(d18:2/23:0)
TG(15:0/15:0/15:0)
Viscutin
7E,9E,11−Dodecatrienyl
Tamarixetin
Luteolin
Enkephalin
6−Methoxykaempferol
Myricetin
Epothilone
14,15−HxA3−D(11S)
methyl
Ikarisoside
Clarithromycin
Artonin
Protorifamycin
Hexacosanedioic
Docosanedioic
bacteriohopane−32,33,34,35−tetrol
Matricin
26,26,26,27,27,27−hexafluoro−25−hydroxy−23,23,24,24−tetradehydrovitamin
17−HOME(9Z)
9−pentadecen−1−ol
Cytochalasin
Tanariflavanone
alpha−licanic
(3S)−7−hydroxy−2',3',4',5',8−pentamethoxyisoflavan
Hippuristanolide
5alpha,6beta−dihydroxycholestanol

b

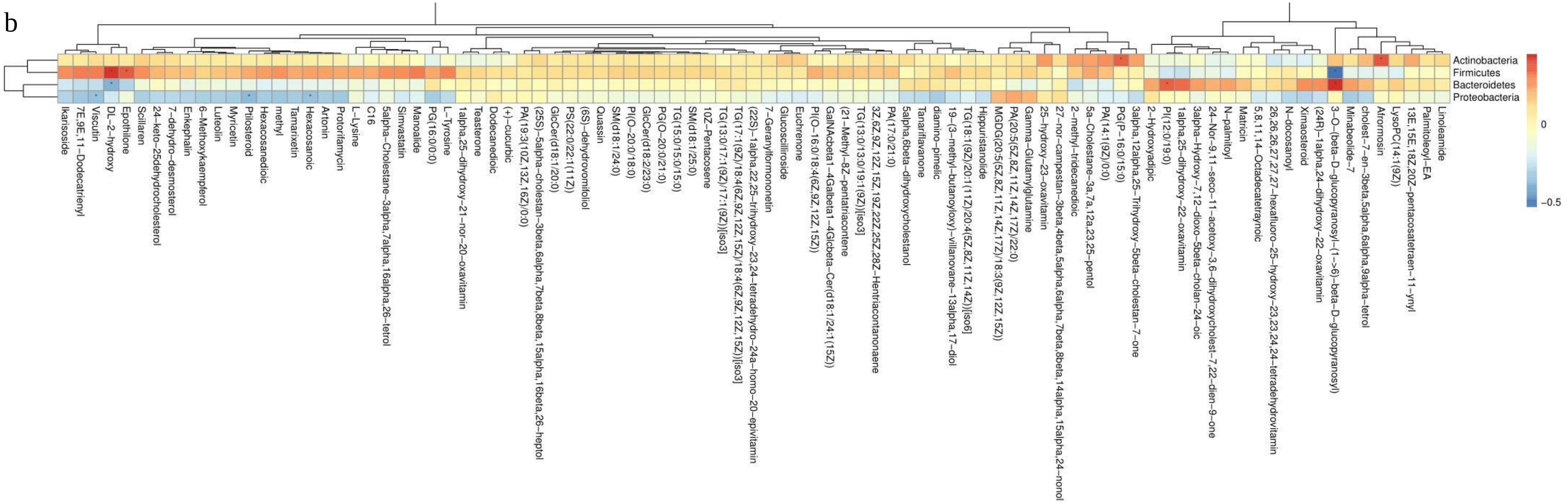


Supplementary Figure 2: Correlation heatmap of differentially enriched metabolites and gut microbiota phylum between the newly diagnosed T2DM and IGR group (a); Correlation heatmap of differentially enriched metabolites and gut microbiota phylum between the newly diagnosed IGR and NGT group (b)

Note: Each column in the graph represents a metabolite, each row represents a microbiota phylum, the colour in the graph indicates the Pearson coefficient between the bacterial phylum and the metabolite, and the red colour indicates positive correlation. The green colour is representative negative correlation. The darker colour indicate the greater the correlation. * is represents a significant correlation (*P* <0.05).

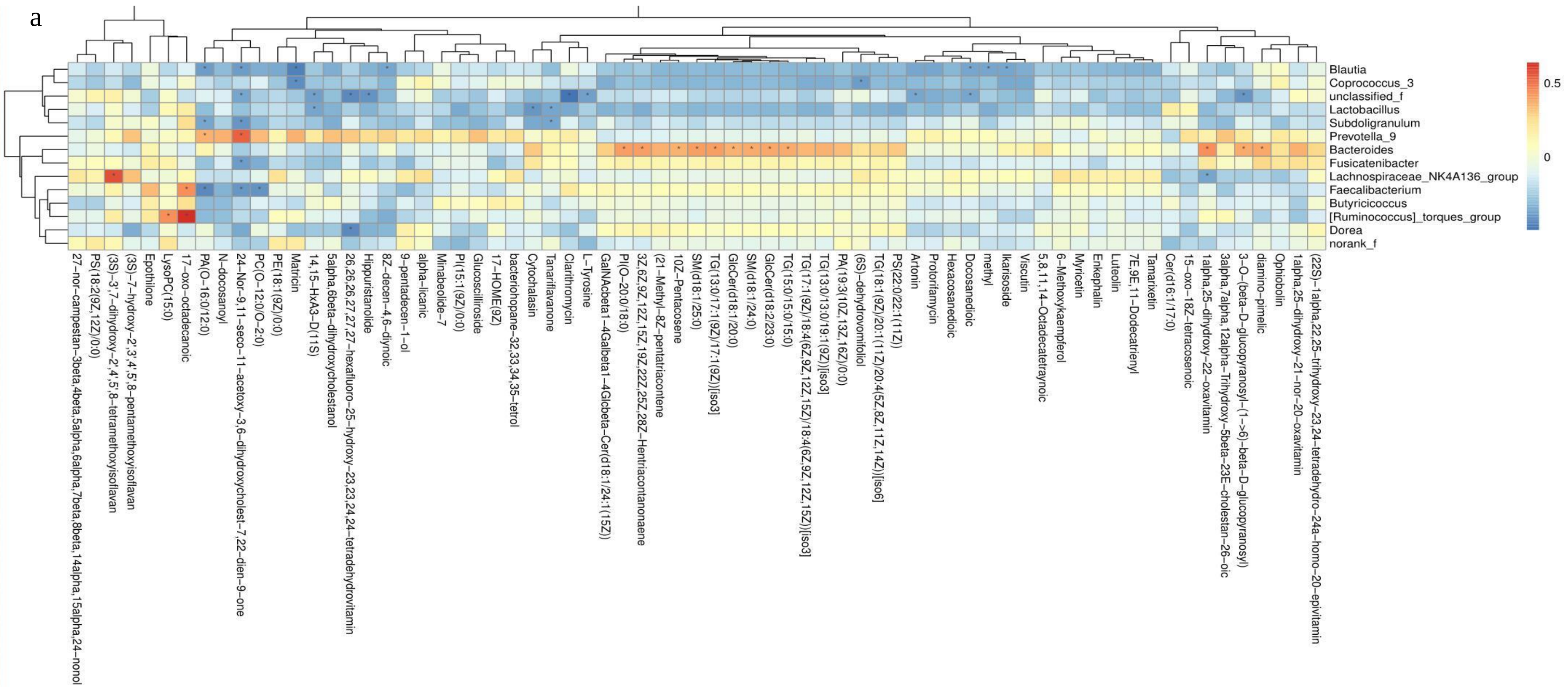
a
Blautia
Coprococcus_3
unclassified_f
Lactobacillus
Subdoligranulum
Prevotella_9
Bacteroides
Fusicatenibacter
Lachnospiraceae_NK4A136_group
Faecalibacterium
Butyricicoccus
[Ruminococcus]_torques_group
Dorea
norank_f
0.5
0
27−nor−campestan−3beta,4beta,5alpha,6alpha,7beta,8beta,14alpha,15alpha,24−nonol
PS(18:2(9Z,12Z)/0:0)
(3S)−3',7−dihydroxy−2',4',5',8−tetramethoxyisoflavan
(3S)−7−hydroxy−2',3',4',5',8−pentamethoxyisoflavan
Epothilone
LysoPC(15:0)
17−oxo−octadecanoic
PA(O−16:0/12:0)
N−docosanoyl
24−Nor−9,11−seco−11−acetoxy−3,6−dihydroxycholest−7,22−dien−9−one
PC(O−12:0/O−2:0)
PE(18:1(9Z)/0:0)
Matricin
14,15−HxA3−D(11S)
5alpha,6beta−dihydroxycholestanol
26,26,26,27,27,27−hexafluoro−25−hydroxy−23,23,24,24−tetradehydrovitamin
Hippuristanolide
8Z−decen−4,6−diynoic
9−pentadecen−1−ol
alpha−licanic
Minabeolide−7
PI(15:1(9Z)/0:0)
Glucoscilliroside
17−HOME(9Z)
bacteriohopane−32,33,34,35−tetrol
Cytochalasin
Tanariflavanone
Clarithromycin
L−Tyrosine
GalNAcbeta1−4Galbeta1−4Glcbeta−Cer(d18:1/24:1(15Z))
PI(O−20:0/18:0)
3Z,6Z,9Z,12Z,15Z,19Z,22Z,25Z,28Z−Hentriacontanonaene
(21−Methyl−8Z−pentatriacontene
10Z−Pentacosene
SM(d18:1/25:0)
TG(13:0/17:1(9Z)/17:1(9Z))[iso3]
GlcCer(d18:1/20:0)
SM(d18:1/24:0)
GlcCer(d18:2/23:0)
TG(15:0/15:0/15:0)
TG(17:1(9Z)/18:4(6Z,9Z,12Z,15Z)/18:4(6Z,9Z,12Z,15Z))[iso3]
TG(13:0/13:0/19:1(9Z))[iso3]
PA(19:3(10Z,13Z,16Z)/0:0)
(6S)−dehydrovomifoliol
TG(18:1(9Z)/20:1(11Z)/20:4(5Z,8Z,11Z,14Z))[iso6]
PS(22:0/22:1(11Z))
Artonin
Protorifamycin
Hexacosanedioic
Docosanedioic
methyl
Ikarisoside
Viscutin
5,8,11,14−Octadecatetraynoic
6−Methoxykaempferol
Myricetin
Enkephalin
Luteolin
7E,9E,11−Dodecatrienyl
Tamarixetin
Cer(d16:1/17:0)
15−oxo−18Z−tetracosenoic
1alpha,25−dihydroxy−22−oxavitamin
3alpha,7alpha,12alpha−Trihydroxy−5beta−23E−cholestan−26−oic
3−O−(beta−D−glucopyranosyl−(1−>6)−beta−D−glucopyranosyl)
diamino−pimelic
Ophiobolin
1alpha,25−dihydroxy−21−nor−20−oxavitamin
(22S)−1alpha,22,25−trihydroxy−23,24−tetradehydro−24a−homo−20−epivitamin

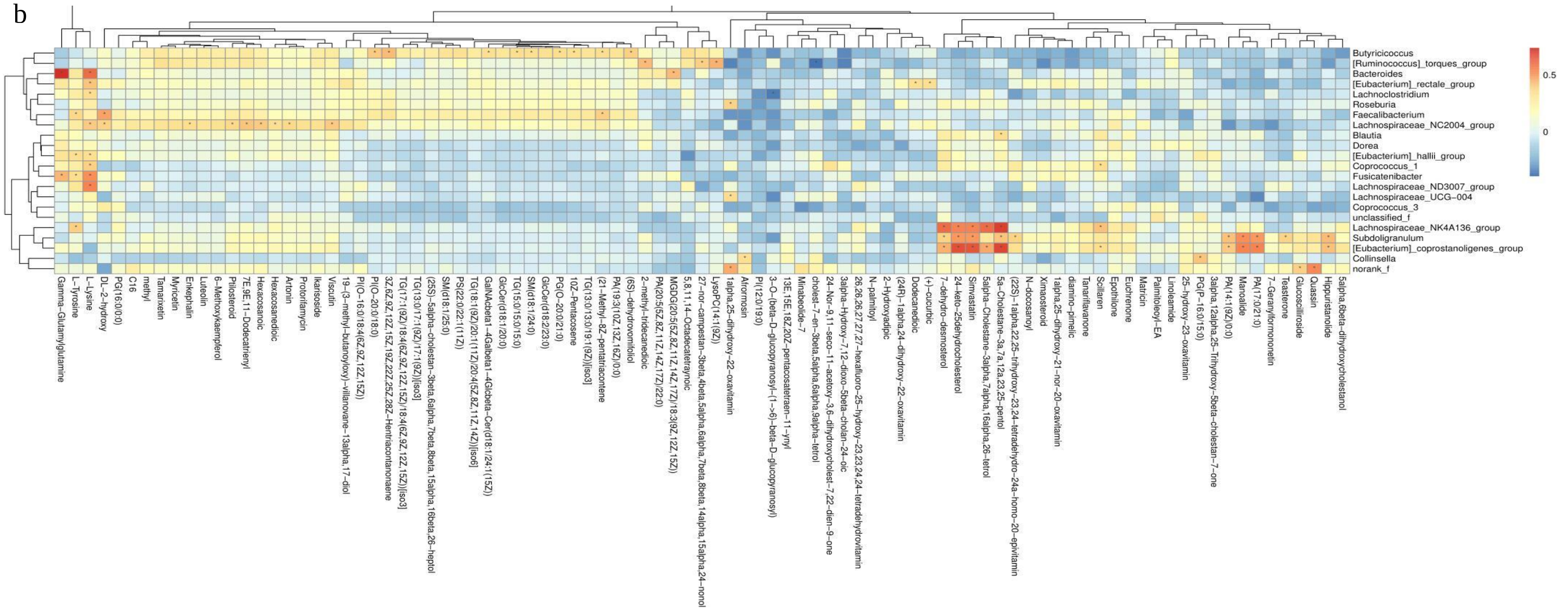


Supplementary Figure 3: Correlation heatmap of differentially enriched metabolites and gut microbiota genus between the newly diagnosed T2DM and IGR group (a); Correlation heatmap of differentially enriched metabolites and gut microbiota genus between the newly diagnosed IGR and NGT group (b).

Note: Each column in the graph represents a metabolite, each row represents a microbiota genus, the colour in the graph indicates the Pearson coefficient between the bacterial genus and the metabolite, and the red colour indicates positive correlation. The green colour is representative negative correlation. The darker colour indicate the greater the correlation. * is represents a significant correlation ($P$ <0.05).

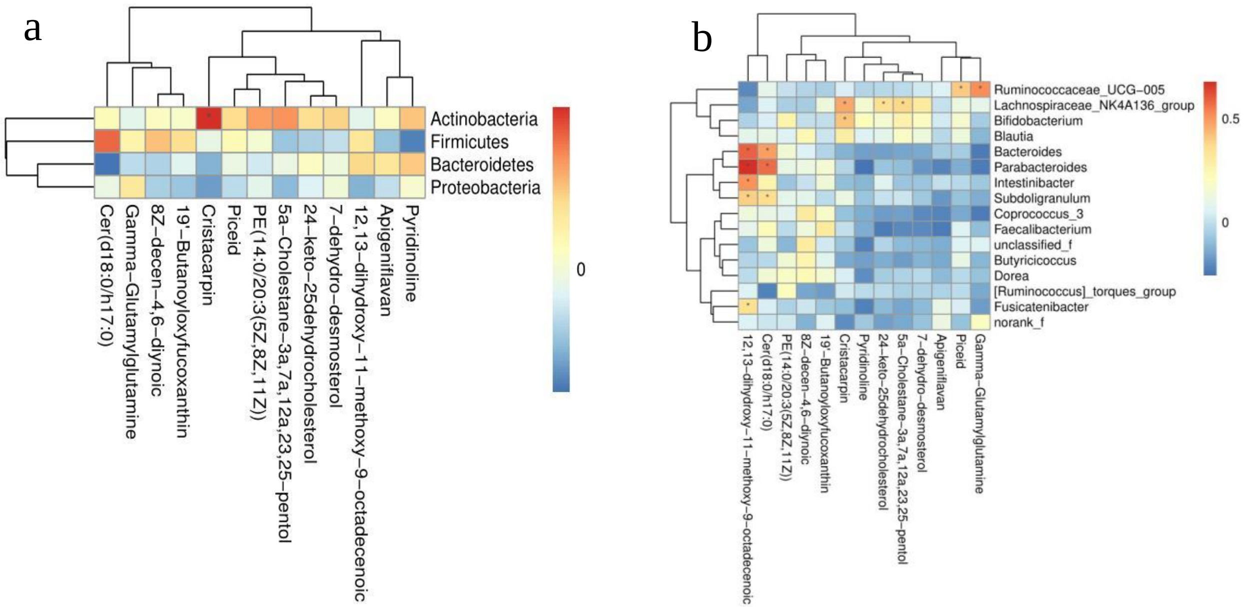


Supplementary Figure 4: Correlation heatmap of differentially enriched metabolites and gut microbiota phylum level between the newly diagnosed T2DM and NGT group (a), Correlation heatmap of differentially enriched metabolites and gut microbiota genus level between the newly diagnosed T2DM and NGT group (b).

Supplementary Table 1: Annotated differentially enriched metabolites between the newly diagnosed T2DM and IGR groups

| Metabolites | m/z | RT (min) | Compound ID | Formula | VIP value | p value | FC (T2DM/ IGR) | Pathways | superclass | class | subclass |
|---|---|---|---|---|---|---|---|---|---|---|---|
| L-Tyrosine | 180.07 | 1.14 | HMDB00158 | C9H11NO3 | 1.36 | 0.00 | 1.64 | Phenylalanine, tyrosine and tryptophan biosynthesis | Organic acids and derivatives | Carboxylic acids and derivatives | Amino acids, peptides, and analogues |
| Epothilone A | 476.25 | 5.84 | LMPK04000040 | C26H39NO6S | 4.02 | 0.03 | 0.79 | Type I polyketide structures | Lipids and lipid-like molecules | Polyketides | Macrolides and lactone polyketides |
| Anhydrorhodovibrin | 565.44 | 6.68 | LMPR01070135 | C41H58O | 1.86 | 0.01 | 3.27 | Metabolic pathways,Carotenoid biosynthesis | Lipids and lipid-like molecules | Prenol lipids | Isoprenoids |
| Protorifamycin I | 657.34 | 11.13 | LMPK05000007 | C35H45NO10 | 1.71 | 0.00 | 0.78 | Biosynthesis of ansamycins,Biosynthesis of antibiotics | Phenylpropanoids and polyketides | Coumarins and derivatives | Furanocoumarins |
| Pimelic acid | 159.07 | 1.16 | LMFA01170051 | C7H12O4 | 1.04 | 0.05 | 0.15 | Metabolic pathways | Lipids and lipid-like molecules | Fatty acyls | Fatty acids and conjugates |
| LysoPC(15:0) | 480.31 | 6.26 | HMDB10381 | C23H48NO7P | 5.44 | 0.05 | 0.36 | Glycerophospholipid metabolism,Choline metabolism in cancer | Lipids and lipid-like molecules | Glycerophospholipids | Glycerophosphocholines |
| Matricin | 613.30 | 2.35 | LMPR0103410003 | C17H22O5 | 3.11 | 0.02 | 0.64 | Unknown | Organoheterocyclic compounds | Lactones | Gamma butyrolactones |
| bacteriohopane-32,33,34,35-tetrol | 564.50 | 7.83 | LMPR04000004 | C35H62O4 | 1.80 | 0.05 | 2.06 | Unknown | Lipids and lipid-like molecules | Prenol lipids | Hopanoids |
| 5alpha,6beta-dihydroxycholestanol | 443.35 | 5.23 | LMST01010052 | C27H48O3 | 1.41 | 0.02 | 1.33 | Unknown | Lipids and lipid-like molecules | Sterol lipids | Sterols |
| Cytochalasin A | 460.25 | 2.12 | LMPK11000001 | C29H35NO5 | 1.24 | 0.02 | 1.26 | Unknown | Lipids and lipid-like molecules | Polyketides | Cytochalasins |
| (6S)-dehydrovomifoliol | 221.12 | 3.27 | LMPR0103050009 | C13H18O3 | 1.08 | 0.00 | 0.74 | Unknown | Lipids and lipid-like molecules | Prenol lipids | Isoprenoids |

Supplementary Table 2: Annotated differentially enriched metabolites between the newly diagnosed T2DM and NGT groups

| Metabolites | m/z | RT (min) | Compound ID | Formula | VIP value | p value | Pathways | superclass | class | subclass |
|---|---|---|---|---|---|---|---|---|---|---|
| Cristacarpin | 355.15 | 3.25 | LMPK12070114 | C21H22O5 | 3.85 | 0.03 | Unknown | Phenylpropanoids and polyketides | Isoflavonoids | Furanoisoflavonoids |
| Piceid | 373.13 | 2.35 | LMPK13090012 | C20H22O8 | 3.49 | 0.01 | Unknown | Phenylpropanoids and polyketides | Stilbenes | Stilbene glycosides |
| 7-dehydro-desmosterol | 383.33 | 4.95 | LMST01010121 | C27H42O | 2.63 | 0.00 | Steroid biosynthesis,Metabolic pathways | Lipids and lipid-like molecules | Sterol lipids | Sterols |
| PE(14:0/20:3) | 712.49 | 9.32 | HMDB08836 | C39H72NO8P | 1.78 | 0.02 | Unknown | Lipids and lipid-like molecules | Glycerophospholipids | Glycerophosphoethanolamines |
| Gamma-Glutamylglutamine | 274.10 | 0.64 | HMDB11738 | C10H17N3O6 | 1.19 | 0.03 | Unknown | Organic acids and derivatives | Carboxylic acids and derivatives | Amino acids, peptides, and analogues |

Supplementary Table 3: Annotated differentially enriched metabolites between the IGR and NGT groups

| Metabolites | m/z | RT (min) | Compound ID | Formula | VIP value | p value | FC (IGR/ NGT) | Pathways | superclass | class | subclass |
|---|---|---|---|---|---|---|---|---|---|---|---|
| Dodecanedioic acid | 229.14 | 3.27 | HMDB00623 | C12H22O4 | 4.60 | 0.01 | 2.43 | Biosynthesis of secondary metabolites,alpha-Linolenic acid metabolism | Lipids and lipid-like molecules | Fatty acyls | Fatty acids and conjugates |
| Matricin | 613.30 | 2.35 | LMPR0103410003 | C17H22O5 | 3.05 | 0.01 | 1.68 | unknown | Organoheterocyclic compounds | Lactones | Gamma butyrolactones |
| L-Tyrosine | 180.07 | 1.14 | HMDB00158 | C9H11NO3 | 2.67 | 0.00 | 0.47 | | Organic acids and derivatives | Carboxylic acids and derivatives | Amino acids, peptides, and analogues |
| LysoPC(14:1) | 464.28 | 6.06 | HMDB10380 | C22H44NO7P | 2.59 | 0.01 | 2.92 | Choline metabolism in cancer,Glycerophospholipid metabolism | Lipids and lipid-like molecules | Glycerophospholipids | Glycerophosphocholines |
| 7-dehydro-desmosterol | 383.33 | 4.95 | LMST01010121 | C27H42O | 2.23 | 0.01 | 0.58 | Steroid biosynthesis,metabolic pathways | Lipids and lipid-like molecules | Sterol lipids | Sterols |
| 5alpha,6beta-dihydroxycholestanol | 443.35 | 5.23 | LMST01010052 | C27H48O3 | 2.15 | 0.01 | 0.66 | unknown | Lipids and lipid-like molecules | Sterol lipids | Sterols |
| Epothilone A | 476.25 | 5.84 | LMPK04000040 | C26H39NO6S | 1.96 | 0.04 | 1.24 | Type I polyketide structures | Lipids and lipid-like molecules | Polyketides | Macrolides and lactone polyketides |
| Protorifamycin I | 657.34 | 11.13 | LMPK05000007 | C35H45NO10 | 1.90 | 0.00 | 1.29 | Biosynthesis of ansamycins,Biosynthesis of antibiotics | Phenylpropanoids and polyketides | Coumarins and derivatives | Furanocoumarins |
| (+)-cucurbic acid | 211.13 | 3.27 | LMFA02020013 | C12H20O3 | 1.62 | 0.00 | 1.91 | unknown | Lipids and lipid-like molecules | Sterol lipids | Sterols |
| Teasterone | 447.35 | 5.63 | LMST01030121 | C28H48O4 | 1.61 | 0.01 | 0.37 | Biosynthesis of secondary metabolites,Brassinosteroid biosynthesis,Metabolic pathways | Lipids and lipid-like molecules | Fatty acyls | Octadecanoids |
| Manoalide | 415.25 | 3.85 | LMPR0105020001 | C25H36O5 | 1.59 | 0.01 | 0.40 | unknown | Lipids and lipid-like molecules | Sterol lipids | Sterols |
| Gamma-Glutamylglutamine | 274.10 | 0.64 | HMDB11738 | C10H17N3O6 | 1.50 | 0.00 | 0.43 | unknown | Organic acids and derivatives | Carboxylic acids and derivatives | Amino acids, peptides, and analogues |
| (6S)-dehydrovomifoliol | 221.12 | 3.27 | LMPR0103050009 | C13H18O3 | 1.25 | 0.01 | 1.35 | unknown | Lipids and lipid-like molecules | Prenol lipids | Isoprenoids |
| Estrone 3-glucuronide | 445.19 | 1.40 | LMST05010011 | C24H30O8 | 1.22 | 0.00 | 0.09 | Steroid hormone biosynthesis | Lipids and lipid-like molecules | Steroids and steroid derivatives | Steroidal glycosides |
| Anhydrorhodovibrin | 565.44 | 6.68 | LMPR01070135 | C41H58O | 1.17 | 0.02 | 0.37 | Carotenoid biosynthesis,Metabolic | Lipids and lipid-like molecules | Prenol lipids | Isoprenoids |

| | | | | | | | | | | | |
|---|---|---|---|---|---|---|---|---|---|---|---|
| | | | | | | | | pathways | | | |
| Quassin | 389.20 | 2.68 | LMPR0106110002 | C22H28O6 | 1.11 | 0.02 | 0.71 | unknown | Lipids and lipid-like molecules | Prenol lipids | Terpene lactones |
| Scillaren A | 715.33 | 2.30 | LMST01130005 | C36H52O13 | 1.03 | 0.04 | 0.74 | unknown | Lipids and lipid-like molecules | Sterol lipids | Sterols |
| 2-Hydroxyadipic acid | 161.05 | 0.64 | LMFA01170049 | C6H10O5 | 1.01 | 0.04 | 1.97 | unknown | Lipids and lipid-like molecules | Fatty acyls | Fatty acids and conjugates |
| L-Lysine | 145.10 | 0.55 | HMDB00182 | C6H14N2O2 | 1.01 | 0.00 | 0.46 | | Organic acids and derivatives | Carboxylic acids and derivatives | Amino acids, peptides, and analogues |
